# The usefulness of obesity and lipid-related indices to predict the presence of Non-alcoholic fatty liver disease

**DOI:** 10.1186/s12944-021-01561-2

**Published:** 2021-10-10

**Authors:** Guotai Sheng, Song Lu, Qiyang Xie, Nan Peng, Maobin Kuang, Yang Zou

**Affiliations:** 1 Department of Cardiology, Jiangxi Provincial People’s Hospital Affiliated to Nanchang University, Nanchang, PR China 330006; 2 From the Jiangxi Provincial Cardiovascular Institute, Jiangxi Provincial People’s Hospital Affiliated to Nanchang University, Nanchang, PR China 330006

**Keywords:** TyG index-related parameters, obesity and lipid-related indices, non-alcoholic fatty liver disease, receiver operating characteristic, general population

## Abstract

**Background:**

Conicity index, body-shape index, lipid accumulation product (LAP), waist circumference (WC), triglyceride, triglyceride-glucose (TyG) index, hepatic steatosis index (HSI), waist-to-height ratio (WHtR), TyG index-related parameters (TyG-WHtR, TyG-BMI, TyG-WC), body mass index (BMI), visceral adiposity index, triglyceride to high-density lipoprotein cholesterol ratio and body roundness index have been reported as reliable markers of non-alcoholic fatty liver disease (NAFLD). However, there is debate about which of the above obesity and lipid-related indices has the best predictive performance for NAFLD risk.

**Methods:**

This study included 6870 female and 7411 male subjects, and 15 obesity and lipid-related indices were measured and calculated. NAFLD was diagnosed by abdominal ultrasound. The area under the curve (AUC) of 15 obesity and lipid-related indices were calculated by receiver operating characteristic (ROC) analysis.

**Results:**

Among the 15 obesity and lipid-related indices, the TyG index-related parameters had the strongest association with NAFLD. ROC analysis showed that except for ABSI, the other 14 parameters had high predictive value in identifying NAFLD, especially in female and young subjects. Most notably, TyG index-related parameters performed better than other parameters in predicting NAFLD in most populations. In the female population, the AUC of TyG-WC for predicting NAFLD was 0.9045, TyG-BMI was 0.9084, and TyG-WHtR was 0.9071. In the male population, the AUC of TyG-WC was 0.8356, TyG-BMI was 0.8428, and TyG-WHtR was 0.8372. In addition, BMI showed good NAFLD prediction performance in most subgroups (AUC>0.8).

**Conclusions:**

Our data suggest that TyG index-related parameters, LAP, HSI, BMI, and WC appear to be good predictors of NAFLD. Of these parameters, TyG index-related parameters showed the best predictive potential.

**Supplementary Information:**

The online version contains supplementary material available at 10.1186/s12944-021-01561-2.

## Background

Non-alcoholic fatty liver disease (NAFLD) is recognized as an important risk factor for peripheral vascular disease, diabetes, kidney disease, and cardio-cerebrovascular disease, and its most significant feature is hepatic steatosis [1–3]. In addition to hepatic steatosis, NAFLD also includes pathological changes such as nonalcoholic steatohepatitis and cirrhosis characterized by liver cell injury, fibrosis activation, and inflammation of lobular necrosis [3, 4]. NAFLD is not a benign, static disease, and without intervention, patients with hepatic steatosis will progress to liver fibrosis over time [5].

In recent decades, with changes in diet, the prevalence of obesity, and the increasing lack of physical exercise, the proportion of patients with NAFLD has increased rapidly [6]. According to the global survey report of NAFLD in 2016, the prevalence of NAFLD diagnosed by imaging exceeded 25%, including 31.79% in the Middle East, 30.45% in South America, 24.13% in North America, 27.37% in Asia, and 23.71% in Europe [7]. The increasing burden of NAFLD is a serious global public health challenge [7, 8].

However, at present, the gold standard for NAFLD diagnosis is still based on liver biopsy results [9]. This invasive examination method is extremely inconvenient for routine health surveillance and large-scale epidemiological investigation of the general population and runs counter to the Helsinki Declaration. Therefore, many studies have focused on identifying simple and effective alternatives for epidemiological investigations and extensive population health surveillance to facilitate early identification of patients most likely to develop NAFLD [10, 11]. In this context, a number of simple anthropometric indicators, biochemical indicators, and some combination of indicators, and even complex digital models have been developed to assess NAFLD risk. Among them, obesity and lipid-related indices are the most effective markers for predicting NAFLD and are widely used in epidemiological studies, including triglyceride (TG), conicity index (COI), visceral adiposity index (VAI), triglyceride-glucose (TyG) index, body roundness index (BRI), body mass index (BMI), hepatic steatosis index (HSI), waist circumference (WC), body-shape index (ABSI), waist-to-height ratio (WHtR), lipid accumulation product (LAP), triglyceride to high-density lipoprotein cholesterol (TG/HDL-C) ratio, and TyG index-related parameters (TyG-WHtR, TyG-BMI, TyG-WC) [12–21]. At present, there are very few comparative studies on the prediction of NAFLD by obesity and lipid-related indices. The conclusions of several existing studies are controversial, and do not include TyG index-related parameters [21–23]. According to some recent studies, TyG index-related parameters are promising new indicators for predicting NAFLD [14, 24]. It is critical to further compare TyG index-related parameters with other obesity and lipid-related indices and identify more useful biomarkers for predicting NAFLD. Therefore, the present study was designed to evaluate the best obesity and lipid-related indices for predicting NAFLD through an epidemiological survey of 14251 from the general population who underwent health screening.

## Methods

### Study design and data

The subjects of this study were from the NAGALA study, the design and implementation of which is described elsewhere [25]. In short, the NAGALA study, officially launched in 1994, is a population-based and ongoing cohort study of the general population who participated in a general health checkup at Murakami Memorial Hospital after the project was initiated. The NAGALA study aimed to assess NAFLD and diabetes by collating and analyzing medical data from the general population. The available data for research was shared by Fukui et al. with the Dryad database [26]. According to the terms of use of the database, different researchers can use the data for post-hoc analysis according to different research hypotheses, so that the data can play a greater role.

The present study was designed to further evaluate the most useful obesity and lipid-related indices for predicting NAFLD based on data from the large population in the NAGALA cohort. This study conducted a cross-sectional design according to the new research purpose, extracted the data of 20944 subjects who took part in the physical examination project from 2004 to 2015 in the NAGALA study, and set the following exclusion criteria according to the new research hypothesis: (a) Male subjects who had excessive alcohol intake during the baseline interview defined as consuming more than or equal to 210 g per week and female who consumed more than or equal to 140 g per week [27]. (b) Subjects diagnosed with alcoholic fatty liver, viral hepatitis, or diabetes at baseline. (c) Subjects with impaired fasting glucose at baseline visits. (d) Subjects taking drugs at the time of baseline examination. (e) Subjects with missing covariates. Finally, 14251 subjects who met the criteria were included in this study. Informed consent for the use of data was approved by subjects in previous studies [25], and the Murakami Memorial Hospital Research Ethics Committee authorized the NAGALA study. Since this study was a post-hoc analysis of the NAGALA study, there was no need to apply for ethical approval again. The whole study process follows the Declaration of Helsinki.

### Data collection and measurement

As previously mentioned [25], the subjects' general clinical data and lifestyle factors were recorded using standardized self-completed questionnaires, including habit of exercise, sex, height, diastolic/systolic blood pressure (D/SBP), age, WC, weight, and smoking/drinking habits. The drinking status was divided into three categories by asking the subjects' weekly alcohol intake: no or little (<40 g/w), light or moderate (40–139 g/w and 140–209 g/w). The smoking status was divided into nonsmokers, former smokers, and current smokers at baseline. Habit of exercise were defined as subjects regularly participating in any type of exercise more than once a week. Hematological samples were collected in the morning after fasting for at least 8 hours. Gamma-glutamyl transferase (GGT), triglyceride (TG), fasting blood glucose (FPG), high-density lipoprotein cholesterol (HDL-C), alanine aminotransferase (ALT), hemoglobin A1c (HbA1c), total cholesterol (TC), and aspartate aminotransferase (AST) were analyzed and determined by an automatic analyzer according to the standard method.

**Fig. 1:**
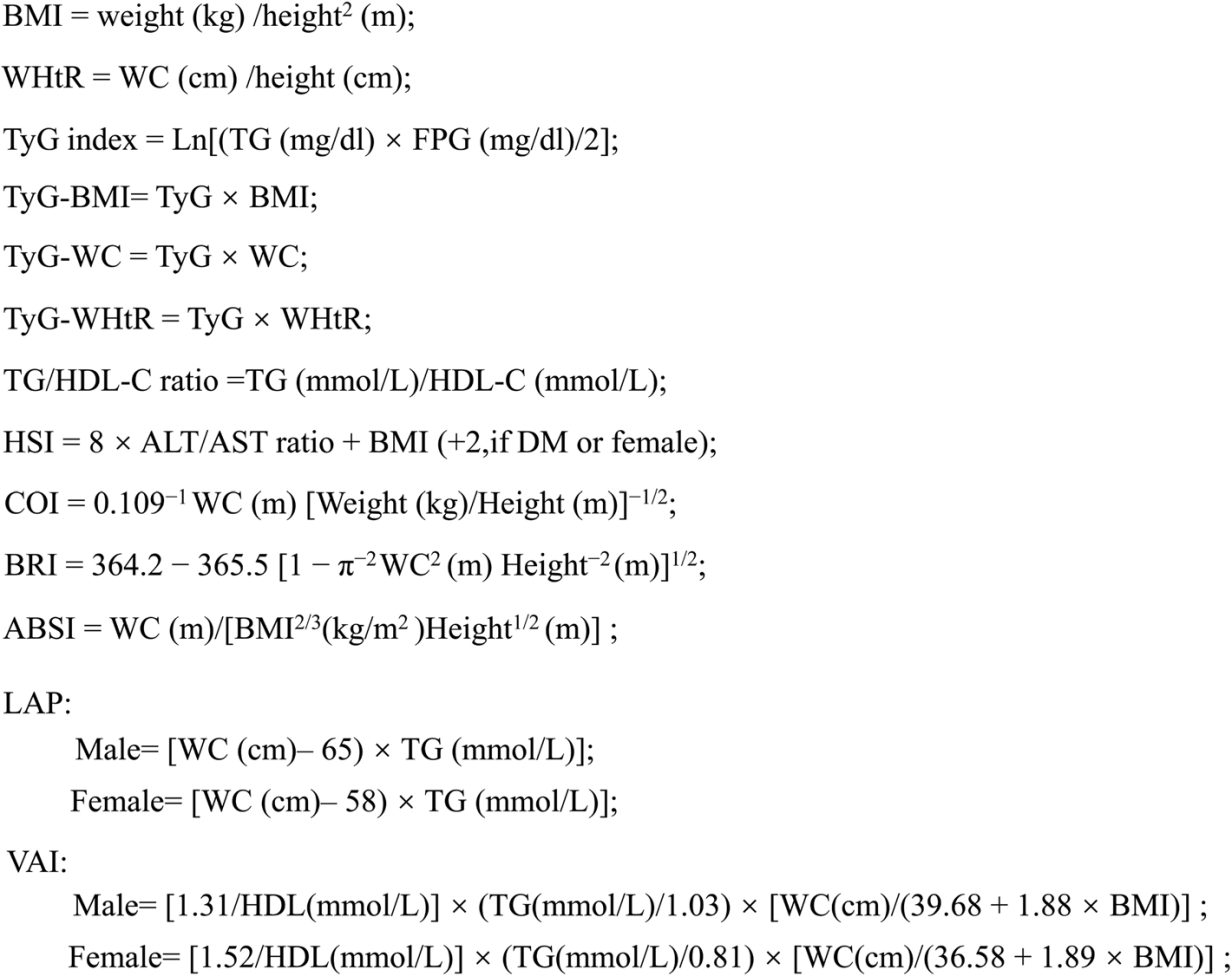
Formulas for calculating obesity and lipid-related indices

The formulas for calculating obesity and lipid-related indices are shown in Fig. 1 [13–21].

### Diagnosis of NAFLD

NAFLD diagnosis was based on the detection of hepatic steatosis by ultrasound while excluding drugs, viruses, or alcohol as the cause. Liver ultrasonography was performed by trained technicians, and experienced gastroenterologists examined the sonograms without knowing the subjects' clinical information and biochemical results. NAFLD diagnosis was based on the following four ultrasonic manifestations with a score of 0–6: liver brightness (0–4 points), hepatorenal echo contrast (0–4 points), vascular blurring (0–1 points), and deep attenuation (0–2 points). If the final score is greater than or equal to 2 points, a diagnosis was made [28].

### Statistical analysis

Given the significant differences in body composition between males and females, all analyses were stratified according to sex. All data were analyzed using Empower (R) version 2.20 and R language version 3.4.3. Categorical variables were represented by numbers (%) and compared using the Pearson χ2 test. For continuous variables, the QQ plot and Shapiro–Wilk test were first used to check their distribution patterns. Continuous variables consistent with or approximately normal distribution were represented by the mean (standard deviation), while those with skewed distribution were represented by the median (interquartile range) and compared by nonparametric test or Student’s t-test. Multiple logistic regression models were established to calculate the odds ratio (OR) and corresponding 95% confidence intervals (CI) for NAFLD with different obesity and lipid-related indices. To allow direct comparison of OR values, 15 obesity and lipid-related indices were converted into Z-scores. The multivariate model adjusted for potential non-collinear confounding variables, such as GGT, age, drinking status, HbA1c, TC, smoking status, HDL-C, habit of exercise, and DBP (Supplementary Table 1). Additionally, to compare the predictive power of 15 obesity and lipid-related indices for NAFLD and determine the best threshold for each parameter, the ROC curve was used to analyze each parameter and find the point at which the sum of sensitivity + specificity was maximized to determine the best threshold for each parameter. Area under the curve (AUC) was interpreted according to the following criteria: <0.5, not useful; 0.5–0.7, poor; 0.7–0.9, good; and 0.9–1.0, excellent.

## Results

### Characteristics of the study subjects

The study included a total of 6870 female and 7411 male subjects, with mean ages of 43.27 and 44.78 years, respectively. Among these subjects, 478 females (3.35%) and 2029 males (14.24%) were diagnosed with NAFLD. Table 1 describes the general clinical characteristics, biochemical characteristics, and characteristics of obesity and lipid-related indices based on whether the study subjects were diagnosed with NAFLD. Some significant differences were observed between the groups with and without NAFLD. In both males and females, people with NAFLD had higher age, WHtR, LAP, AST, weight, BRI, BMI, TyG-WHtR, VAI, ALT/AST ratio, TyG index, HbA1c, TyG-BMI, ABSI, TG/HDL-C ratio, GGT, ALT, WC, TC, TG, FPG, S/DBP, HSI, TyG-WC, and COI. Additionally, among male subjects, there were also significant differences in physical exercise habits between NAFLD patients and healthy individuals, as NAFLD patients tended not to exercise.

**Table 1:** Characteristics of the study subjects with and without NAFLD

	Female			Male		
	Non-NAFLD	NAFLD	*P*-value	Non-NAFLD	NAFLD	*P*-value
No of subjects	6362	478		5382	2029	
Age, years	42.89 (8.72)	47.64 (8.29)	<0.001	43.71 (9.27)	44.11 (8.20)	<0.001
Weight, kg	51.86 (7.06)	63.17 (9.97)	<0.001	64.65 (8.34	74.30 (10.56)	<0.001
Height, cm	158.37 (5.38)	157.04 (5.28)	<0.001	170.89 (6.04)	170.62 (5.94)	0.084
BMI, kg/m^2^	20.67 (2.57)	25.58 (3.57)	<0.001	22.12 (2.42)	25.48 (3.02)	<0.001
WC, cm	70.80 (7.30)	83.27 (8.86)	<0.001	77.99 (6.77)	86.62 (7.37)	<0.001
WHtR	0.45 (0.05)	0.53 (0.06)	<0.001	0.46 (0.04)	0.51 (0.04)	<0.001
TyG index	7.68 (0.54)	8.35 (0.53)	<0.001	8.14 (0.55)	8.64 (0.54)	<0.001
TyG-BMI	159.09 (25.66)	213.85 (34.25)	<0.001	180.37 (26.19)	220.34 (31.27)	<0.001
TyG-WC	544.58 (76.07)	696.06 (91.31)	<0.001	635.94 (81.00)	748.77 (84.35)	<0.001
TyG-WHtR	3.44 (0.49)	4.44 (0.59)	<0.001	3.72 (0.48)	4.39 (0.48	<0.001
ALT, IU/L	13.00 (11.00-17.00)	19.00 (15.00-26.00)	<0.001	18.00 (14.00-23.00)	29.00 (22.00-41.00)	<0.001
AST, IU/L	16.00 (13.00-19.00)	18.00 (15.00-22.00)	<0.001	17.00 (14.00-21.00)	21.00 (17.00-26.00)	<0.001
ALT/AST ratio	0.86 (0.71-1.00)	1.12 (0.89-1.37)	<0.001	1.07 (0.87-1.31)	1.43 (1.17-1.76)	<0.001
GGT, IU/L	12.00 (9.00-14.00)	15.00 (12.00-20.00)	<0.001	17.00 (14.00-24.00)	24.00 (18.00-35.00)	<0.001
HDL-C, mmol/L	1.66 (0.38)	1.38 (0.34)	<0.001	1.35 (0.35)	1.14 (0.25)	<0.001
TC, mmol/L	5.05 (0.86)	5.56 (0.92)	<0.001	5.06 (0.84	5.41 (0.85	<0.001
TG, mmol/L	0.54 (0.40-0.77)	1.02 (0.73-1.38)	<0.001	0.80 (0.58-1.16)	1.32 (0.91-1.86)	<0.001
FPG, mmol/L	4.94 (4.72-5.22)	5.27 (5.00-5.55)	<0.001	5.22 (5.00-5.50)	5.44 (5.16-5.66)	<0.001
HbA1c, %	5.17 (0.32)	5.42 (0.33)	<0.001	5.13 (0.31)	5.27 (0.33)	<0.001
SBP, mmHg	108.42 (13.77)	120.71 (16.04)	<0.001	116.04 (13.16)	124.04 (14.46)	<0.001
DBP, mmHg	67.00 (9.48)	75.11 (10.22) 74.50	<0.001	72.88 (9.32)	78.44 (10.08)	<0.001
TG/HDL-C	0.33 (0.22-0.50)	0.75 (0.50-1.11)	<0.001	0.61 (0.40-0.98)	1.19 (0.76-1.82)	<0.001
HSI	29.80 (3.58)	36.74 (4.76)	<0.001	31.05 (4.16)	37.43 (5.12)	<0.001
VAI	0.57 (0.39-0.88)	1.37 (0.89-2.05)	<0.001	0.74 (0.47-1.21)	1.49 (0.95-2.31)	<0.001
LAP	6.15 (3.27-11.14)	23.34 (15.09-36.34)	<0.001	9.92 (5.29-17.72)	27.81 (17.07-42.42)	<0.001
ABSI	0.07 (0.07-0.08)	0.08 (0.07-0.08)	<0.001	0.08 (0.07-0.08)	0.08 (0.07-0.08)	<0.001
BRI	2.33 (1.88-2.92)	3.82 (3.21-4.61)	<0.001	2.56 (2.12-3.04)	3.42 (2.98-3.97)	<0.001
COI	1.14 (0.07)	1.21 (0.07)	<0.001	1.16 (0.06)	1.21 (0.05)	<0.001
Habit of exercise			0.335			<0.001
No	5351 (84.11%)	410 (85.77%)		4300 (79.90%)	1720 (84.77%)	
Yes	1011 (15.89%)	68 (14.23%)		1082 (20.10%)	309 (15.23%)	
Drinking status			0.004			<0.001
no or little	5986 (94.09%)	465 (97.28%)		3731 (69.32%)	1623 (79.99%)	
light	376 (5.91%)	13 (2.72%)		1096 (20.36%)	273 (13.45%)	
moderate	0 (0%)	0 (0%)		555 (10.31%)	133 (6.55%)	
Smoking status			0.664			0.067
Non	5609 (88.16%)	427 (89.33%)		1952 (36.27%)	758 (37.36%)	
Former	382 (6.00%)	24 (5.02%)		1538 (28.58%)	615 (30.31%)	
Current	371 (5.83%)	27 (5.65%)		1892 (35.15%)	656 (32.33%)	

Values were expressed as mean (SD) or medians (quartile interval) or n (%). Abbreviations: NAFLD: Nonalcoholic fatty liver disease; BMI: body mass index; WC: Waist circumference; WHtR: waist-to-height ratio; TyG index: triglyceride-glucose index; TyG-BMI: triglyceride glucose-body mass index; TyG-WC: triglyceride glucose-waist circumference; TyG-WHtR: triglyceride glucose- waist-to-height ratio; ALT: alanine aminotransferase; AST: aspartate aminotransferase; GGT: gamma-glutamyl transferase; HDL-C: high-density lipoprotein cholesterol; TC: total cholesterol; TG: triglyceride; TG/HDL-C: triglyceride to high-density lipoprotein cholesterol ratio; HbA1c: hemoglobin A1c; FPG: fasting plasma glucose; SBP: systolic blood pressure; DBP: Diastolic blood pressure; HIS: hepatic steatosis index; VAI: visceral adiposity index; LAP: lipid accumulation product; ABSI: body-shape index; BRI: body roundness index; COI: conicity index.

### Associations of 15 obesity and lipid-related indices with NAFLD

Table 2 shows the association between 15 obesity and lipid-related indices and NAFLD risk in males and females. As expected, 15 obesity and lipid-related indices were positively correlated with NAFLD before and after model adjustment. It is worth noting that NAFLD was strongly correlated with TyG-related parameters and HSI in both males and females, and TyG-WHtR was the most strongly correlated with NAFLD among all subjects (OR= 4.47 for men and OR = 5.56 for women, both *P* < 0.05). In addition, some anthropometric indicators, such as WC, BMI, and WHtR, were also strongly correlated with NAFLD in males. However, VAI, TG/HDL-C ratio, ABSI and TG were weakly correlated with NAFLD in the whole population.

**Table 2:** Association of NAFLD with the level of 15 obesity and lipid-related indices

	Female		Male	
	Univariable	Multivariable	Univariable	Multivariable
BMI (Per SD)	4.23 (3.80, 4.71)	3.14 (2.79, 3.55)	4.56 (4.16, 4.73)	3.35 (3.06, 3.67)
WC (Per SD)	4.86 (4.31, 5.49)	3.31 (2.89, 3.79)	4.96 (4.54, 5.41)	3.55 (3.22, 3.92)
WHtR (Per SD)	3.99 (3.60, 4.43)	2.85 (2.53, 3.20)	4.58 (4.22, 4.98)	3.50 (3.19, 3.85)
TG (Per SD)	3.65 (3.22, 4.13)	1.86 (1.60, 2.18)	2.02 (1.92, 2.14)	1.34 (1.25, 1.43)
TyG (Per SD)	4.35 (3.83, 4.94 )	2.43 (2.06, 2.86)	2.87 (2.68, 3.08)	1.79 (1.63, 1.95)
TyG-BMI (Per SD)	5.96 (5.25, 6.76)	4.62 (3.97, 5.38)	5.50 (5.04, 6.00)	4.31 (3.88, 4.79)
TyG-WC (Per SD)	7.73 (6.66, 8.96)	5.56 (4.65, 6.63)	5.65 (5.16, 6.18)	4.47 (4.00, 5.00)
TyG-WHtR (Per SD)	5.81 (5.12, 6.60)	4.53 (3.87, 5.29)	5.18 (4.75, 5.64)	4.37 (3.92, 4.87)
TG/HDL-C ratio (Per SD)	4.42 (3.81, 5.11)	1.80 (1.48, 2.18)	2.03 (1.92, 2.14)	1.28 (1.19, 1.37)
HSI (Per SD)	5.36 (4.74, 6.05)	4.03 (3.52, 4.62)	4.26 (3.95, 4.59)	3.52 (3.24, 3.83)
LAP (Per SD)	5.33 (4.69, 6.07)	3.41 (2.91, 4.00)	3.10 (2.90, 3.31)	2.24 (2.07, 2.43)
VAI (Per SD)	3.01 (2.70, 3.34)	1.61 (.40, 1.86)	2.14 (2.02, 2.27)	1.33 (1.24, 1.43)
ABSI (Per SD)	1.43 (1.32, 1.55)	1.18 (1.08, 1.30)	1.40 (1.31, 1.49)	1.29 (1.19, 1.39)
BRI (Per SD)	3.58 (3.25, 3.94)	2.58 (2.32, 2.88)	4.36 (4.02, 4.73)	3.32 (2.80, 3.22)
COI (Per SD)	2.34 (2.14, 2.56)	1.71 (1.54, 1.90)	2.63 (2.45, 2.83)	2.15 (1.97, 2.34)

Abbreviations: OR: Odds ratios; other abbreviations as in Table ​1.

Adjusted for age, habit of exercise, GGT, TC, HDL-C, HbA1c, smoking status, drinking status and DBP.

### Evaluate the accuracy of obesity and lipid-related indices in predicting NAFLD in the whole population

The accuracy of 15 obesity and lipid-related indices in predicting NAFLD in the whole population were first evaluated by ROC analysis. As shown in Table 3, the accuracy of the TyG index-related parameters, LAP, HSI, BMI, and WC in predicting NAFLD was relatively good. Among them, the AUC of TyG-BMI was the largest (0.8862), with a sensitivity of 0.8381, specificity of 0.7787, and best threshold of 189.6932.

**Table 3:** The best threshold, Positive-LR, Negative-LR, sensitivities, specificities, Youden index, and area under the curve of obesity and lipid-related indices for the screening of NAFLD in the general population

	AUC	95%CI low	95%CI upp	Best threshold	Positive-LR	Negative-LR	Specificity	Sensitivity	Youden index
BMI	0.8577	0.8503	0.8651	22.5521	2.8561	0.2104	0.7015	0.8524	0.5539
WC	0.8610	0.8539	0.8681	79.6500	3.3282	0.2529	0.7571	0.8085	0.5656
WHtR	0.8366	0.8289	0.8442	0.4715	2.6973	0.2392	0.6905	0.8349	0.5254
TG	0.7969	0.7877	0.8061	0.8411	2.4052	0.3385	0.6799	0.7698	0.4497
FPG	0.7111	0.7005	0.7217	5.1902	1.8080	0.4775	0.6073	0.7100	0.3173
TyG	0.8084	0.7996	0.8173	8.2059	2.6571	0.3392	0.7149	0.7575	0.4724
TyG-BMI	0.8862	0.8797	0.8927	189.6932	3.7869	0.2080	0.7787	0.8381	0.6168
TyG-WC	0.8846	0.8782	0.8911	637.8369	3.1606	0.1627	0.7207	0.8827	0.6034
TyG-WHtR	0.8766	0.8700	0.8833	3.8957	3.3328	0.2053	0.7459	0.8468	0.5927
TG/HDL-C ratio	0.8147	0.8060	0.8233	0.6119	2.5289	0.2865	0.6818	0.8046	0.4864
HSI	0.8678	0.8604	0.8752	32.9224	3.5455	0.2557	0.7738	0.8022	0.5760
VAI	0.8000	0.7909	0.8092	0.9306	2.5657	0.3446	0.7049	0.7571	0.462
LAP	0.8659	0.8588	0.8730	13.7281	3.2019	0.2279	0.7404	0.8313	0.5717

Abbreviations: AUC: area under the curve; other abbreviations as in Table ​1.

### Evaluate the accuracy of obesity and lipid-related indices in predicting NAFLD in different sexes

**Fig. 2:**
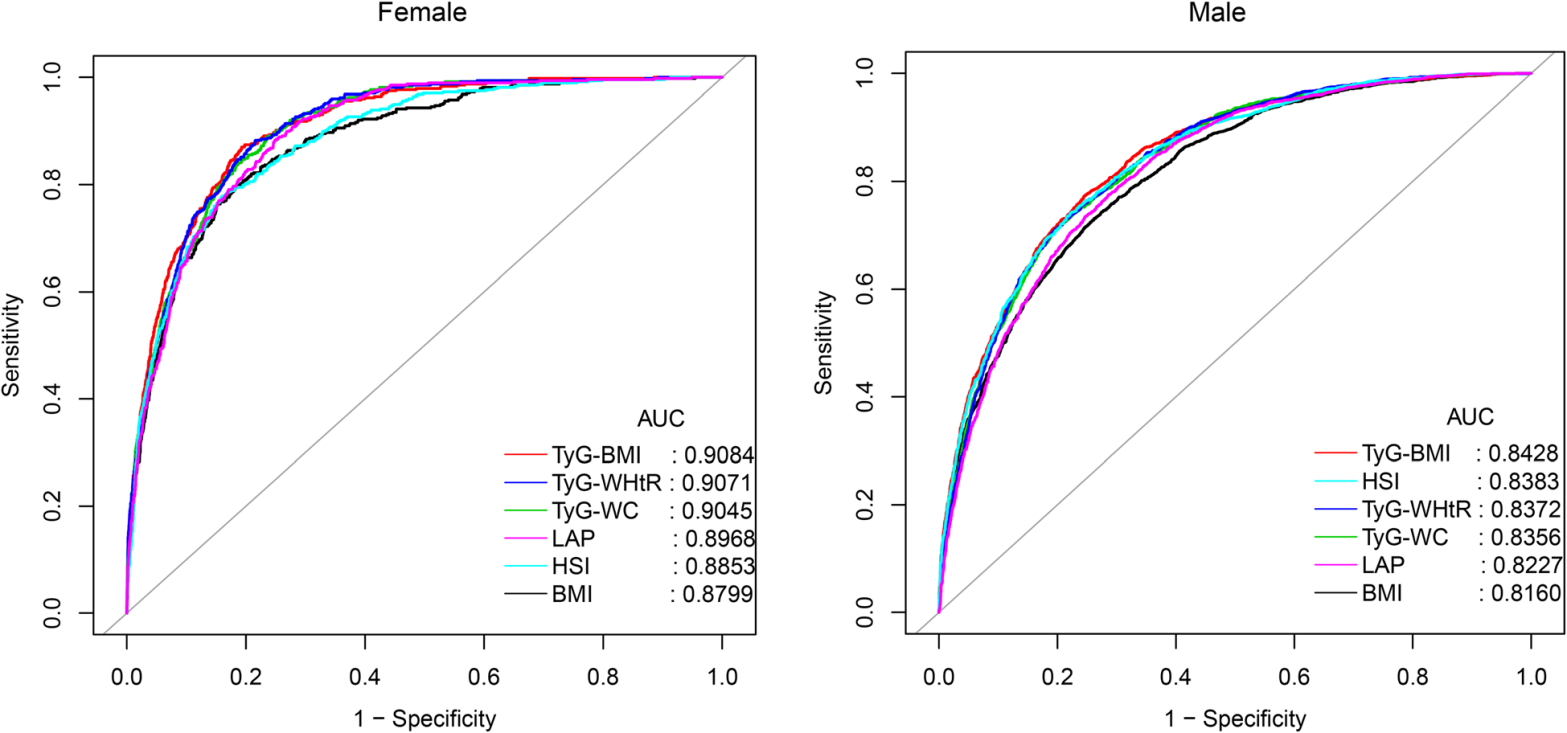
ROC curve analysis of NAFLD-related indicators in females and males. BMI: Body mass index; TyG-BMI: Triglyceride-glucose index-related body mass index; TyG-WHtR: Triglyceride-glucose index-related waist-to-height ratio; TyG-WC: triglyceride-glucose index-related waist circumference; HSI: hepatic steatosis index; LAP: lipid accumulation product; NAFLD: non-alcoholic fatty liver disease

Figure 2 and Table 4 show the results of ROC analysis and the AUC of 15 obesity and lipid-related indices used to predict NAFLD in females and males. The AUCs of all 15 obesity and lipid-related indices were greater than 0.5, indicating that all have certain predictive values for NAFLD.

**Table 4:** The best threshold, Positive-LR, Negative-LR, sensitivities, specificities, and area under the curve of obesity and lipid-related indices for the screening of NAFLD in male and female

	AUC	95%CI low	95%CI upp	Best threshold	Positive-LR	Negative-LR	Specificity	Sensitivity
**Female**								
BMI	0.8799	0.8648	0.8950	22.7565	4.3600	0.2507	0.8177	0.7950
WC	0.8695	0.8545	0.8844	74.2500	2.9640	0.1781	0.7050	0.8745
WHtR	0.8790	0.8647	0.8932	0.4735	3.2918	0.1763	0.7356	0.8703
TG	0.8049	0.7852	0.8246	0.7169	2.6208	0.3206	0.7047	0.7741
TyG	0.8186	0.7998	0.8375	7.9863	2.8028	0.3121	0.7238	0.7741
TyG-BMI	0.9084	0.8964	0.9204	178.7047	4.5125	0.1607	0.8071	0.8703
TyG-WC	0.9045	0.8926	0.9163	595.3694	3.7964	0.1448	0.7658	0.8891
TyG-WHtR	0.9071	0.8954	0.9188	3.8078	4.0878	0.1494	0.7840	0.8828
TG/HDL-C ratio	0.8175	0.7984	0.8366	0.4955	2.9826	0.3327	0.7482	0.7510
HSI	0.8853	0.8707	0.9000	33.0155	4.8780	0.2639	0.8405	0.7782
VAI	0.8281	0.8098	0.8464	0.8228	2.8087	0.2886	0.7177	0.7929
LAP	0.8968	0.8845	0.9092	11.1715	3.5526	0.1559	0.7515	0.8828
ABSI	0.6171	0.5916	o.6426	0.0758	1.4634	0.6814	0.5926	0.5962
BRI	0.8790	0.8647	0.8932	2.8750	2.7918	0.3645	0.7356	0.8703
COI	0.7523	0.7310	0.7735	1.1610	2.1143	0.3940	0.6478	0.7448
**Male**								
BMI	0.8160	0.8055	0.8264	23.5555	2.7918	0.3645	0.7382	0.7309
WC	0.8102	0.7998	0.8207	80.6500	2.4154	0.2946	0.6674	0.8034
WHtR	0.8156	0.8054	0.8257	0.4736	2.4753	0.2886	0.6747	0.8053
TG	0.7367	0.7242	0.7492	1.0669	2.1749	0.4959	0.6997	0.6530
TyG	0.7458	0.7336	0.7581	8.4415	2.3265	0.4851	0.7204	0.6506
TyG-BMI	0.8428	0.8331	0.8525	196.8688	3.1537	0.3015	0.7551	0.7723
TyG-WC	0.8356	0.8257	0.8454	699.2287	3.4331	0.3498	0.7891	0.7240
TyG-WHtR	0.8372	0.8274	0.8469	4.0945	3.4107	0.3442	0.7861	0.7294
TG/HDL-C ratio	0.7499	0.7378	0.7620	0.8398	2.1539	0.4395	0.6731	0.7042
HSI	0.8383	0.8285	0.8481	33.9371	3.3201	0.3314	0.7763	0.7427
VAI	0.7565	0.7445	0.7684	1.1117	2.3351	0.4548	0.7101	0.6770
LAP	0.8227	0.8126	0.8328	16.2553	2.7094	0.3148	0.7139	0.7753
ABSI	0.5795	0.5653	0.5936	0.0749	1.2173	0.6811	0.4052	0.7240
BRI	0.8156	0.8054	0.8257	2.8767	2.4753	0.2886	0.6747	0.8053
COI	0.7117	0.6992	0.7242	1.1749	1.7526	0.4635	0.5838	0.7294

Abbreviations: AUC: area under the curve; other abbreviations as in Table ​1.

Among females, the AUC of TyG-related parameters were the largest, all of which were more than 0.90, in which TyG-WHtR was 0.9071 (95% CI: 0.8954–0.9188), TyG-BMI was 0.9084 (95% CI: 0.8964–9204), and TyG-WC was 0.9045 (95% CI: 0.8926–0.9163). The best thresholds of TyG-WHtR, TyG-BMI, and TyG-WC for predicting NAFLD were 3.8078, 178.7047, and 595.3694, respectively. Additionally, BMI, WC, WHtR, HSI, LAP, and BRI also had a high predictive performance for NAFLD, and their AUCs were greater than 0.85.

In males, the AUC of WC, BMI, WHtR, TyG-related parameters, HSI, LAP, and BRI was larger, all exceeding 0.8, BMI was 0.8160, WC was 0.8102, WHtR was 0.8156, TyG-WC was 0.8356, TyG-BMI was 0.8428, TyG-WHtR was 0.8372, HSI was 0.8383, LAP was 8227, and BRI was 0.8156. TyG-BMI was the best marker for predicting male NAFLD, and its best threshold was 196.8688. In contrast, ABSI's performance in predicting NAFLD was mediocre in both males and females.

### Evaluate the accuracy of obesity and lipid-related indices in predicting NAFLD in different sex and age groups

Table 5 and Table 6 show the AUCs of 15 obesity and lipid-related indices for predicting NAFLD in females and males at different ages. In the young female population (age 18–30 years), except ABSI and COI, the remaining 13 obesity and lipid-related markers had excellent performance in predicting NAFLD (all AUC>0.9), among which LAP was the best predictor in the young female population (AUC=0.9801). In the middle-aged female population (age 31–45 years), WC, BMI, WHtR, TyG-WC, HSI, TyG-BMI, LAP, TyG-WHtR, and BRI were all excellent predictors of NAFLD, among which the AUC of TyG-BMI was the largest (AUC=0.9436). However, in females over 45 years old, the accuracy of obesity and lipid-related indices in predicting NAFLD decreased. TyG-WHtR was the best marker for predicting NAFLD in middle-aged and elderly females (age 46–60 years: AUC=0.8541), and TyG-BMI was the best marker for predicting NAFLD in older females (age>60 years: AUC=0.8853).

**Table 5:** Areas under the ROC curves for obesity and lipid-related indices as a predictor of nonalcoholic fatty liver disease in female subjects of different ages

	AUC	95%CI low	95%CI upp	Best threshold	Positive-LR	Negative-LR	Specificity	Sensitivity
**Age>60years**								
BMI	0.8424	0.7447	0.9400	23.3334	5.8333	0.1944	0.8571	0.8333
WC	0.7525	0.6356	0.8694	80.2500	3.0196	0.4278	0.7792	0.6667
WHtR	0.7511	0.6357	0.8665	0.5258	0.4716	7.3915	0.8247	0.6111
TG	0.6825	0.5326	0.8325	1.1911	3.2906	0.5347	0.8312	0.5556
TyG	0.6843	0.5423	0.8264	8.5008	3.2906	0.5347	0.8312	0.5556
TyG-BMI	0.8853	0.8080	0.9625	185.5919	4.2778	0.0713	0.7792	0.9444
TyG-WC	0.8045	0.7033	0.9056	666.3764	4.1067	0.3979	0.8377	0.6667
TyG-WHtR	0.8023	0.7014	0.9032	3.9938	2.4889	0.1728	0.6429	0.8889
TG/HDL-C ratio	0.6659	0.5131	0.8188	0.9641	4.5294	0.5620	0.8896	0.5000
HSI	0.8506	0.7547	0.9466	34.3072	9.2685	0.3013	0.9221	0.7222
VAI	0.6685	0.5178	0.8191	1.8359	5.9231	0.5461	0.9156	0.5000
LAP	0.8014	0.7079	0.8950	11.8347	2.1079	0.1007	0.5519	0.9444
ABSI	0.5321	0.3973	0.6669	0.0782	1.4444	0.5556	0.5000	0.7222
BRI	0.7511	0.6357	0.8665	3.8560	3.4856	0.4716	0.8247	0.6111
COI	0.6118	0.4789	0.7448	1.2326	1.9861	0.4365	0.6364	0.7222
**Age 46-60years**								
BMI	0.8198	0.7937	0.8459	22.7522	3.1495	0.3229	0.7605	0.7544
WC	0.8160	0.7913	0.8407	74.7500	2.3102	0.2516	0.6364	0.8399
WHtR	0.8244	0.7998	0.8490	0.4858	2.8061	0.3011	0.7210	0.7829
TG	0.7390	0.7086	0.7694	0.7734	2.0204	0.4100	0.6336	0.7402
TyG	0.7549	0.7256	0.7842	8.1052	2.2148	0.4162	0.6754	0.7189
TyG-BMI	0.8497	0.8276	0.8719	178.7047	2.9930	0.2088	0.7158	0.8505
TyG-WC	0.8494	0.8280	0.8707	607.7065	2.7895	0.2291	0.6989	0.8399
TyG-WHtR	0.8541	0.8331	0.8752	3.8790	2.8579	0.2314	0.7074	0.8363
TG/HDL-C ratio	0.7591	0.7304	0.7877	0.4955	2.1445	0.3788	0.6482	0.7544
HSI	0.8455	0.8226	0.8684	32.8451	3.4263	0.3107	0.7788	0.7580
VAI	0.7708	0.7431	0.7986	0.9154	2.2600	0.3775	0.6693	0.7473
LAP	0.8377	0.8155	0.8598	14.0538	2.9346	0.2961	0.7332	0.7829
ABSI	0.6234	0.5900	0.6567	0.0758	1.4818	0.6110	0.5533	0.6619
BRI	0.8244	0.7998	0.8490	3.0960	2.8061	0.3011	0.7210	0.7829
COI	0.7228	0.6931	0.7525	1.1619	1.7995	0.4134	0.5768	0.7616
**Age 31-45years**								
BMI	0.9311	0.9156	0.9467	23.0613	6.3800	0.1744	0.8670	0.8488
WC	0.9113	0.8927	0.9298	75.3500	4.0862	0.1484	0.7837	0.8837
WHtR	0.9155	0.8975	0.9334	0.4719	4.1807	0.0970	0.7789	0.9244
TG	0.8296	0.7971	0.8621	0.7169	3.3271	0.3228	0.7746	0.7500
TyG	0.8404	0.8090	0.8719	7.9863	3.7081	0.3134	0.7977	0.7500
TyG-BMI	0.9436	0.9280	0.9592	179.6766	6.5608	0.1146	0.8626	0.9012
TyG-WC	0.9354	0.9191	0.9518	595.3694	5.1492	0.1129	0.8239	0.9070
TyG-WHtR	0.9343	0.9172	0.9515	3.8736	7.0991	0.1652	0.8796	0.8547
TG/HDL-C ratio	0.8453	0.8140	0.8767	0.4767	3.6570	0.2807	0.7870	0.7791
HSI	0.9169	0.8957	0.9381	33.1586	6.6599	0.1797	0.8734	0.8430
VAI	0.8542	0.8242	0.8842	0.9040	4.1209	0.2990	0.8166	0.7558
LAP	0.9281	0.9109	0.9454	10.0086	4.1807	0.0970	0.7789	0.9244
ABSI	0.5611	0.5192	0.6029	0.0713	1.1665	0.4503	0.2324	0.8953
BRI	0.9155	0.8975	0.9334	2.8477	.1807	0.0970	0.7789	0.9244
COI	0.7508	0.7166	0.7851	1.1610	2.2617	0.4166	0.6838	0.7151
**Age 18-30years**								
BMI	0.9313	0.8531	1.0000	20.8195	3.9355	0.0000	0.7459	1.0000
WC	0.9584	0.9107	1.0000	72.1000	5.6308	0.0000	0.8224	1.0000
WHtR	0.9606	0.9217	0.9995	0.4619	7.1765	0.0000	0.8607	1.0000
TG	0.9288	0.8679	0.9897	0.7508	9.5065	0.1570	0.9098	0.8571
TyG	0.9391	0.8652	1.0000	8.0897	16.5113	0.1507	0.9481	0.8571
TyG-BMI	0.9575	0.9033	1.0000	160.0894	5.2286	0.0000	0.8087	1.0000
TyG-WC	0.9723	0.9357	1.0000	554.8238	7.4694	0.0000	0.8661	1.0000
TyG-WHtR	0.9742	0.9456	1.0000	3.5509	9.8919	0.0000	0.8989	1.0000
TG/HDL-C ratio	0.9465	0.8972	0.9958	0.4417	6.5357	0.0000	0.8470	1.0000
HSI	0.9157	0.8244	1.0000	29.6667	3.1826	0.0000	0.6858	1.0000
VAI	0.9575	0.9177	0.9972	0.8322	8.3182	0.0000	0.8798	1.0000
LAP	0.9801	0.9512	1.0000	8.4370	9.6316	0.0000	0.8962	1.0000
ABSI	0.6511	0.3609	0.9412	0.0759	3.1497	0.3695	0.7732	0.7143
BRI	0.9606	0.9217	0.9995	2.6718	7.1765	0.0000	0.8607	1.0000
COI	0.8158	0.6292	1.0000	1.1613	4.5074	0.3395	0.8415	0.7143

Abbreviations: AUC: area under the curve; other abbreviations as in Table ​1.

**Table 6:** Areas under the ROC curves for obesity and lipid-related indices as a predictor of nonalcoholic fatty liver disease in male subjects of different ages

	AUC	95%CI low	95%CI upp	Best threshold	Positive-LR	Negative-LR	Specificity	Sensitivity
**Age>60years**								
BMI	0.7965	0.7349	0.8582	23.7420	3.9130	0.3495	0.8175	0.7143
WC	0.7716	0.7118	0.8313	80.8000	2.1176	0.2400	0.5952	0.8571
WHtR	0.7639	0.7062	0.8215	0.4992	2.5205	0.3799	0.7103	0.7302
TG	0.6417	0.5679	0.7154	0.9258	1.5221	0.5755	0.5516	0.6825
TyG	0.6570	0.5853	0.7286	8.1646	1.5312	0.4516	0.4921	0.7778
TyG-BM	0.7913	0.7309	0.8518	194.1812	2.7222	0.3111	0.7143	0.7778
TyG-WC	0.7725	0.7101	0.8348	694.1685	2.4658	0.4022	0.7103	0.7143
TyG-WHtR	0.7679	0.7070	0.8287	3.9308	1.8689	0.1846	0.5159	0.9048
TG/HDL-C ratio	0.6515	0.5781	0.7250	0.8877	1.7882	0.5988	0.6627	0.6032
HSI	0.8381	0.7868	0.8894	31.2974	3.2615	0.2139	0.7421	0.8413
VAI	0.6623	0.5894	0.7351	1.1966	1.9733	0.5876	0.7024	0.5873
LAP	0.7481	0.6848	0.8114	13.4633	1.9065	0.3310	0.5754	0.8095
ABSI	0.5600	0.4805	0.6396	0.0759	1.2022	0.4638	0.2738	0.8730
BRI	0.7639	0.7062	0.8215	3.3445	2.5205	0.3799	0.7103	0.7302
COI	0.6557	0.5837	0.7277	1.1898	1.4752	0.3964	0.4405	0.8254
**Age 46-60years**								
BMI	0.7855	0.7663	0.8047	23.5546	2.5148	0.4098	0.7196	0.7051
WC	0.7809	0.7616	0.8002	81.2500	2.1572	0.3190	0.6295	0.7992
WHtR	0.7859	0.7673	0.8045	0.4736	2.0365	0.2452	0.5786	0.8581
TG	0.7066	0.6845	0.7287	1.1121	1.9329	0.5332	0.6665	0.6447
TyG	0.7164	0.6946	0.7381	8.4415	1.9561	0.4908	0.6525	0.6798
TyG-BMI	0.8148	0.7968	0.8329	201.7871	3.2018	0.3671	0.7767	0.7149
TyG-WC	0.8081	0.7899	0.8264	711.4515	3.2917	0.3769	0.7862	0.7037
TyG-WHtR	0.8109	0.7929	0.8290	4.1294	2.8242	0.3320	0.7320	0.7570
TG/HDL-C ratio	0.7202	0.6986	0.7418	0.7396	1.7295	0.3940	0.5462	0.7848
HSI	0.8133	0.7953	0.8313	33.1439	2.8738	0.3466	0.7415	0.7430
VAI	0.7260	0.7046	0.7473	0.9306	1.7734	0.3761	0.5535	0.7918
LAP	0.7923	0.7736	0.8110	17.8721	2.4420	0.3504	0.6894	0.7584
ABSI	0.5696	0.5449	0.5942	0.0772	.2425	0.7989	0.5467	0.5632
BRI	0.7859	0.7673	0.8045	2.8766	2.0365	0.2452	0.5786	0.8581
COI	0.6828	0.6605	0.7051	1.2056	1.8572	0.5954	0.6794	0.5955
**Age 31-45years**								
BMI	0.8337	0.8209	0.8466	23.4488	2.8721	0.3226	0.7343	0.7631
WC	0.8274	0.8145	0.8403	80.6500	2.6899	0.2950	0.7056	0.7918
WHtR	0.8385	0.8260	0.8509	0.4713	2.8416	0.2866	0.7208	0.7934
TG	0.7521	0.7361	0.7681	1.0669	2.4375	0.4773	0.7333	0.6500
TyG	0.7609	0.7452	0.7766	8.3729	2.3647	0.4404	0.7092	0.6877
TyG-BMI	0.8580	0.8461	0.8699	189.6580	2.6993	0.1844	0.6757	0.8754
TyG-WC	0.8514	0.8393	0.8635	697.6885	3.8683	0.3453	0.8142	0.7189
TyG-WHtR	0.8566	0.8447	0.8684	3.8936	2.8551	0.2393	0.7092	0.8303
TG/HDL-C ratio	0.7657	0.7503	0.7811	0.8216	2.3282	0.4163	0.6947	0.7108
HSI	0.8543	0.8421	0.8664	34.5292	3.7709	0.2989	0.7981	0.7615
VAI	0.7725	0.7574	0.7877	1.0975	2.5612	0.4343	0.7340	0.6812
LAP	0.8395	0.8270	0.8519	13.4323	2.5826	0.2280	0.6721	0.8467
ABSI	0.5880	0.5696	0.6063	0.0746	1.2682	0.6462	0.4312	0.7213
BRI	0.8385	0.8260	0.8509	2.8360	2.8416	0.2866	0.7208	0.7934
COI	0.7364	0.7205	0.7522	1.1727	1.9949	0.4534	0.6454	0.7074
**Age 18-30years**								
BMI	0.8222	0.7488	0.8955	24.4551	3.9070	0.3590	0.8193	0.7059
WC	0.8288	0.7622	0.8954	80.3500	3.2500	0.3077	0.7647	0.7647
WHtR	0.8426	0.7797	0.9054	0.4636	3.3793	0.2333	0.7563	0.8235
TG	0.8352	0.7719	0.8985	0.7959	2.8194	0.2108	0.6975	0.8529
TyG	0.8480	0.7887	0.9073	8.1808	3.3220	0.2346	0.7521	0.8235
TyG-BMI	0.8663	0.8019	0.9307	197.9225	5.2000	0.2759	0.8529	0.7647
TyG-WC	0.8799	0.8216	0.9381	656.3063	4.1087	0.2552	0.8067	0.7941
TyG-WHtR	0.8861	0.8291	0.9430	4.0029	6.0667	0.2692	0.8739	0.7647
TG/HDL-C ratio	0.8591	0.8046	0.9136	0.6008	2.7821	0.1313	0.6723	0.9118
HSI	0.8689	0.8141	0.9237	31.6663	2.5591	0.0000	0.6092	1.0000
VAI	0.8636	0.8089	0.9182	0.9282	4.0213	0.2565	0.8025	0.7941
LAP	0.8814	0.8218	0.9409	18.9616	7.6667	0.3548	0.9118	0.6765
ABSI	0.6422	0.5513	0.7332	0.0734	1.5680	0.3717	0.4748	0.8235
BRI	0.8426	0.7797	0.9054	2.7017	3.3793	0.2333	0.7563	0.8235
COI	0.7818	0.7098	0.8537	1.1398	2.3605	0.2303	0.6387	0.8529

Abbreviations: AUC: area under the curve; other abbreviations as in Table ​1.

In males, a trend similar to that of females was observed. Compared with young and middle-aged males, obesity and lipid-related indices were less accurate in predicting NAFLD in males over 45 years old. TyG-WHtR was the best predictor of NAFLD in young males (age 18–330 years: AUC=0.8861). TyG-BMI was the best predictor of NAFLD in males aged 31 to 60 years (AUC=0.8580 for males aged 31–45 years, AUC=0.8148 for males aged 46–60 years). However, in older males (age>60 years), HSI was the best marker for predicting NAFLD (AUC=0.8381).

## Discussion

This study assessed the ability of 15 commonly used, non-invasive obesity and lipid-related indices to predict NAFLD risk in the general population. The accuracy of ABSI in predicting individual NAFLD risk was limited in both males and females, while the other 14 markers showed better predictive performance for NAFLD, especially in females. Of mention, although there were some differences in the predictive performance of the 15 obesity and lipid-related indices among different populations, TyG index-related parameters were superior to other parameters in predicting NAFLD in most populations. Therefore, TyG-related parameters may be the best choice for NAFLD risk screening indicators in the general population.

Past studies have shown that obesity, metabolic disorders, and environmental factors all contribute to the occurrence and development of NAFLD. However, with the rapid development of society, changes in lifestyle, dietary structure, and the prevalence of obesity, the prevalence of NAFLD is increasing rapidly, bringing a series of adverse consequences [6–8]. Therefore, it is an urgent task to screen vulnerable groups for NAFLD as soon as possible. Because liver biopsy-based tests are invasive, expensive, and time-consuming, noninvasive methods are being widely studied as alternative indicators [10, 11]. Simple measurements such as WC, BMI, WHtR, and blood lipids have an independent correlation with NAFLD [12, 16, 17]. These findings were verified in this study. Compared with WC, WHtR, and TG, BMI was a better predictor of NAFLD. In addition, it is worth mentioning that in this study, the AUC of BMI in most subgroups was greater than 0.8, which means that BMI had a good predictive performance in most populations. Although the incorporation of TyG significantly improves the predictive value of NAFLD in most subgroups, considering the simplicity and convenience of BMI measurement, it does not require additional laboratory measurements, so it should also be considered in the general population.

TyG index is a combination of FPG and TG. Previous studies reported that the index can be used as a substitute marker for IR in the clinic [29–31]. Additionally, it can effectively identify NAFLD and evaluate the risk of NAFLD in females [13, 32]. This finding was further verified in the present study. ROC analysis found that the AUC of TyG index for predicting NAFLD was 0.8186 in females and 0.7458 in males. Additionally, the TyG index has better predictive performance in the young population (age 18–30 years, AUC=0.9391 for females; AUC=0.8480 for males). TyG index-related parameters are the combined parameters of the TyG index with WC, BMI, and WHtR, which were first reported by Ko et al. [33]. They pointed out that TyG index-related parameters had the highest AUC value for predicting IR compared to visceral obesity indicators, lipid parameters, lipid ratios, and adipokines. Subsequent studies showed that TyG index-related parameters were used to predict non-obese, overweight, and obese people's NAFLD better than TyG alone [14, 24, 33]. This study expands the sample size from a previous study and found that TyG index-related parameters have excellent prediction performance in most populations.

TG/HDL-C is the ratio of TG and HDL-C. Similar to the TyG index, the TG/HDL-C ratio can distinguish IR from NAFLD, which has been widely popularized in the clinic [15, 34]. According to Ko et al., the TG/HDL-C ratio is a better predictor of IR than lipid markers and adipokines alone, but its AUC was lower than that of TyG-BMI, VAI, TyG index, LAP, and TyG-WC [32]. Similar results were found in predicting NAFLD in this study, which may be closely related to IR [1, 35].

HSI is a NAFLD prediction model developed by Lee et al [18]. It is a combination of liver enzymes and BMI, and was confirmed in a large number of studies to have excellent predictive performance in predicting NAFLD [36, 37]. According to a recent report by Lin et al., HSI has better prediction performance for NAFLD than BMI, WHtR, LAP, BRI, COI, VAI, TyG index, waist-hip ratio, body adiposity index (BAI) and abdominal volume index (AVI) [21]. However, it is not clear whether TyG index-related parameters are better than HSI and other obesity and lipid-related indices in predicting NAFLD. In this context, the predictive performance of 15 common obesity and lipid-related indices for NAFLD were compared in this study. The results showed that HSI did have better NAFLD identification ability than other parameters, but TyG index-related parameters were better predictors of NAFLD than HSI. In a follow-up study, the Procino team analyzed the predictive value of HSI, WC, fatty liver index, BMI, waist/height^0.5^, AVI, WHtR, and BRI for NAFLD. Their findings contradicted the research of Lin et al., who found that the best indicator of NAFLD screening was AVI, not HSI [22]. Additionally, in a recent study, Zhang et al. evaluated the predictive value of relative fat mass, WC, ABSI, WHtR, COI, ponderal index, BMI, and LAP for NAFLD in the elderly; their study showed that LAP was the best marker of these parameters for predicting NAFLD [23]. This study confirms the conclusion of Zhang et al., and in further analysis, it was found that LAP was superior to the TyG index, WHtR, ABSI, COI, BMI, TG/HDL-C ratio, WC, VAI, TG, and BRI in both males and females. Additionally, it is worth noting that LAP was the best predictor of NAFLD for young females (age 18–30 years, AUC=0.9801). Compared with these previous studies, this study considered more obesity and lipid-related indices, as well as TyG index-related parameters, which have been widely considered recently [14, 24]. In general, TyG index-related parameters may be the best choice for NAFLD risk screening in the general population, whether male or female, whether young, middle-aged or elderly. More importantly, the indicators that make up the TyG index-related parameters are clinically easy to obtain and affordable, which brings great convenience for the prevention and treatment of NAFLD.

In the correlation analysis, the researchers calculated the OR value and 95% CI of the corresponding NAFLD risk after Z-conversion of 15 obesity and lipid-related indices. The results of the study were similar to the results of the ROC analysis. Among the 15 parameters, TyG index-related parameters had the strongest correlation with NAFLD risk, both before and after model adjustment. Although many parameters in this study have a strong correlation with NAFLD and the accuracy of predicting NAFLD was good, the TyG index-related parameters were best.

In this study, the best thresholds of TyG index-related parameters in different sex and different age groups were calculated by ROC analysis, in which the best thresholds of TyG-WHtR, TyG-WC, and TyG-BMI were 3.8078, 595.3694, and 178.7047 in females, respectively and 4.0945, 699.2287, and 196.8688 in males, respectively. Two previous studies also provided data for reference; in the study by Huang et al., the best threshold for TyG-BMI to predict NAFLD in non-obese people was 183.8263 [24]. Khamseh's team reported that the best threshold for NAFLD corresponding to TyG-WC in overweight and obese individuals was 876 [14], which was significantly higher than the best threshold recommended in this study. In view of the obvious differences among the study subjects, only a brief description and report of these results are provided as a reference for subsequent studies.

### Study strength and limitations

Several positive effects should be noted. First, the biggest advantage of this study is that of the 15 obesity and lipid-related indices, TyG index-related parameters had the highest accuracy for predicting NAFLD. Second, this study is based on data analysis of a large sample, and the conclusion can be regarded as relatively reliable. Finally, the study was stratified by sex and age to identify the best parameters and thresholds for predicting NAFLD for different populations. These results provide a reliable reference for precision treatment.

The study has some limitations. First, this study lacks general measurement information such as hip/neck circumference. To our knowledge, the new index combining the TyG index with hip/neck circumference was recently found to have high diagnostic value for IR [38]. IR is the main mediating factor in the pathogenesis of NAFLD [1], and the combination of the TyG index and hip/neck circumference may have excellent performance in the prediction of NAFLD. In addition, due to the lack of hip circumference, AVI and BAI cannot be calculated, so it was not possible to further evaluate the difference in NAFLD between AVI, BAI, and other obesity and lipid-related indices. Second, the diagnosis of NAFLD was made only on the basis of ultrasound. Although the current ultrasound diagnosis has high sensitivity and specificity, it is undeniable that nearly 30% of mild fatty liver may be missed [39], which means that the true prevalence rate of NAFLD in this study may be higher. Third, because the dataset analyzed comes from a public database, the dataset provided in the database cannot be updated, which lacks some parameters used to calculate the score of non-invasive fibrosis. Therefore, unfortunately, staging information on liver fibrosis cannot be provided in the current study. Fourth, the study used data from the Japanese population, so its conclusions may not apply to other ethnic groups. Finally, the research design may be a limitation. The cross-sectional design adopted in this study restricts us from explaining the causal correlation of these variables.

## Conclusion

TyG index-related parameters may be the best choice for NAFLD risk screening in the general population. Considering that the calculation of the TyG index-related parameters is simple and convenient, it is suggested that the TyG index-related parameters should be included in the NAFLD risk assessment list as a marker of focus. The findings of this study provide more comprehensive and referential information for the prevention and treatment of NAFLD and add strong evidence for the non-invasive evaluation of NAFLD.

## Supplementary Information

**Additional file 1.**

## Data Availability

The datasets that support the conclusions of this article can be found in the DRYAD repository.

## Abbreviations

NAFLD: non-alcoholic fatty liver disease
WHtR: waist-to-height ratio
WC: waist circumference
BMI: Body mass index
TG: triglyceride
TyG: triglyceride-glucose
TyG-WC: triglyceride-glucose index-related waist circumference
TyG-BMI: triglyceride-glucose index-related body mass index
TyG-WHtR: triglyceride-glucose index-related waist-to-height ratio
HSI: hepatic steatosis index
VAI: visceral adiposity index
LAP: lipid accumulation product
ABSI: body-shape index
BRI: body roundness index
COI: conicity index
ALT: Alanine aminotransferase
AST: aspartate aminotransferase
GGT: gamma-glutamyl transferase
HDL-C: high-density lipoprotein cholesterol
TC: total cholesterol
TG: triglyceride
HbA1c: hemoglobin A1c
FPG: fasting blood glucose
OR: odds ratio
CI: confidence intervals
ROC: receiver operating characteristic curve
AUC: area under the curve
TG/HDL-C: triglyceride to high-density lipoprotein
IR: insulin resistance
S/DBP: systolic/diastolic blood pressure
