# Supplementary material for: The usefulness of obesity and lipid-related indices to predict the presence of Non-alcoholic fatty liver disease
Source: Lipids Health Dis. 2021 Oct 10;20:134. doi: 10.1186/s12944-021-01561-2 (PMC8502416; doi:10.1186/s12944-021-01561-2)
Supplement: Supplementary file 1 — Additional file 1. [file 12944_2021_1561_MOESM1_ESM.docx]

Supplementary Table 1: Collinearity diagnostics steps.

|  | Step 1 | Step 2 | Step 3 | Step 4 | Step 5 | Step 6 | Step 7 | Step 8 | Step 9 | Step 10 | Step 11 | Step 12 | Step 13 | Step 14 | Step 15 | Step 16 |
| --- | --- | --- | --- | --- | --- | --- | --- | --- | --- | --- | --- | --- | --- | --- | --- | --- |
| Sex | Inf | NA | NA | NA | NA | NA | NA | NA | NA | NA | NA | NA | NA | NA | NA | NA |
| Age | 1.4 | 1.4 | 1.4 | 1.4 | 1.4 | 1.4 | 1.4 | 1.4 | 1.4 | 1.4 | 1.4 | 1.4 | 1.4 | 1.4 | 1.4 | 1.4 |
| BMI | Inf | 2692.1 | 1963.9 | 1824.7 | 1494.7 | NA | NA | NA | NA | NA | NA | NA | NA | NA | NA | NA |
| WC | 8614.2 | 8614.2 | 3855.2 | 1449.1 | 1396.2 | 1394.4 | NA | NA | NA | NA | NA | NA | NA | NA | NA | NA |
| ALT | 16.2 | 16.2 | 16.2 | 16.2 | 16.2 | 16.2 | 16.1 | 16.1 | 16.1 | 16 | 16 | 16 | 16 | NA | NA | NA |
| AST | 11 | 11 | 11 | 10.9 | 10.9 | 10.9 | 10.9 | 10.9 | 10.9 | 10.8 | 10.8 | 10.8 | 10.8 | 1.2 | 1.2 | 1.2 |
| ALT/AST ratio | Inf | 64.1 | 64.1 | 63.5 | 56 | 51.1 | 51.1 | 48.6 | 46.2 | 5.8 | 5.8 | 5.7 | 5.7 | 1.5 | 1.5 | 1.5 |
| Weight | 567.6 | 567.6 | 461.4 | 453.7 | 430.9 | 382.3 | 171.3 | 125.7 | 96.7 | 62.1 | NA | NA | NA | NA | NA | NA |
| Height | 204.2 | 204.2 | 115.6 | 105.7 | 105.7 | 104.6 | 63.4 | 60.6 | 44.9 | 33.5 | 2.2 | 2 | 2 | 2 | 1.6 | 1.6 |
| WHtR | 16454.1 | 16454.1 | 2905 | 2226.6 | 1455.5 | 1098.2 | 336.3 | 300.3 | NA | NA | NA | NA | NA | NA | NA | NA |
| Habit of exercise | 1 | 1 | 1 | 1 | 1 | 1 | 1 | 1 | 1 | 1 | 1 | 1 | 1 | 1 | 1 | 1 |
| GGT | 1.5 | 1.5 | 1.5 | 1.5 | 1.5 | 1.5 | 1.5 | 1.5 | 1.5 | 1.5 | 1.5 | 1.5 | 1.5 | 1.5 | 1.4 | 1.4 |
| TC | 1.6 | 1.6 | 1.6 | 1.6 | 1.6 | 1.6 | 1.6 | 1.6 | 1.6 | 1.6 | 1.6 | 1.6 | 1.6 | 1.6 | 1.6 | 1.6 |
| HDL-C | 2.7 | 2.7 | 2.7 | 2.7 | 2.7 | 2.7 | 2.7 | 2.7 | 2.6 | 2.6 | 2.6 | 2.4 | 2.3 | 2.3 | 2 | 2 |
| TG | 49.3 | 49.3 | 49.3 | 47.6 | 46 | 42.7 | 41.6 | 40.8 | 39 | 39 | 38.7 | 25 | NA | NA | NA | NA |
| FPG | 1.7 | 1.7 | 1.7 | 1.7 | 1.7 | 1.7 | 1.7 | 1.7 | 1.7 | 1.7 | 1.7 | 1.6 | 1.6 | 1.6 | 1.6 | 1.6 |
| TyG | 440.7 | 440.7 | 438.5 | 366.5 | 188.5 | 79.9 | 72.2 | 10.3 | 10.2 | 10.1 | 10.1 | 8.3 | 4.3 | 4.3 | 4.2 | 4.2 |
| TyG-BMI | 1979.8 | 1979.8 | 1947.2 | 1880.8 | 1134.1 | 434 | 391.8 | NA | NA | NA | NA | NA | NA | NA | NA | NA |
| HbA1c | 1.3 | 1.3 | 1.3 | 1.3 | 1.3 | 1.3 | 1.3 | 1.2 | 1.2 | 1.2 | 1.2 | 1.2 | 1.2 | 1.2 | 1.2 | 1.2 |
| Drinking status | 1.2 | 1.2 | 1.2 | 1.2 | 1.2 | 1.2 | 1.2 | 1.2 | 1.2 | 1.2 | 1.2 | 1.2 | 1.2 | 1.2 | 1.2 | 1.2 |
| Smoking status | 1.4 | 1.4 | 1.4 | 1.4 | 1.4 | 1.4 | 1.4 | 1.4 | 1.4 | 1.3 | 1.3 | 1.3 | 1.3 | 1.3 | 1.3 | 1.3 |
| SBP | 5.5 | 5.5 | 5.5 | 5.5 | 5.5 | 5.5 | 5.5 | 5.5 | 5.5 | 5.5 | 5.5 | 5.5 | 5.5 | 5.5 | 5.5 | NA |
| DBP | 5.6 | 5.6 | 5.6 | 5.6 | 5.6 | 5.6 | 5.6 | 5.6 | 5.6 | 5.6 | 5.6 | 5.6 | 5.5 | 5.5 | 5.5 | 1.4 |
| TG/HDL-C ratio | 86.7 | 86.7 | 86.6 | 73.4 | 70.2 | 68.9 | 67.2 | 61.1 | 61.1 | 49.1 | 47.6 | NA | NA | NA | NA | NA |
| TyG-WC | 4253.7 | 4253.7 | 4252.6 | NA | NA | NA | NA | NA | NA | NA | NA | NA | NA | NA | NA | NA |
| TyG-WHtR | 3761.2 | 3761.2 | 3755.4 | 2505.5 | NA | NA | NA | NA | NA | NA | NA | NA | NA | NA | NA | NA |
| HIS | Inf | 152 | 151.7 | 150.1 | 132.1 | 120 | 119.9 | 113.4 | 106.7 | NA | NA | NA | NA | NA | NA | NA |
| VAI | 78.5 | 78.5 | 78.5 | 64.1 | 61.5 | 60 | 58.1 | 51.3 | 51.3 | 37.8 | 36.3 | 13.6 | 8.4 | 8.4 | 3.2 | 3.2 |
| LAP | 54.6 | 54.6 | 54.5 | 45.1 | 38.1 | 24.7 | 21.9 | 17.3 | 14.7 | 14.7 | 14.3 | 13 | 10.8 | 10.7 | NA | NA |
| ABSI | 14773.3 | 14773.3 | 106.7 | 104.5 | 97.9 | 51.3 | 41.8 | 23.7 | 12.6 | 10.9 | 1.6 | 1.6 | 1.6 | 1.6 | 1.5 | 1.5 |
| BRI | 1825.8 | 1825.8 | 202.8 | 202.8 | 192.5 | 186.7 | 185.3 | 180.6 | 49.6 | 43.5 | 5.3 | 5.2 | 4.5 | 4.5 | 2.2 | 2.1 |
| COI | 45051 | 45051 | NA | NA | NA | NA | NA | NA | NA | NA | NA | NA | NA | NA | NA | NA |

VIF = 1/(1-R^2^). Abbreviations as in Table ​1.
